# Methyl 4,6-bis­(4-fluoro­phen­yl)-2-oxo­cyclo­hex-3-ene-1-carboxyl­ate

**DOI:** 10.1107/S1600536810009414

**Published:** 2010-03-17

**Authors:** Hoong-Kun Fun, Madhukar Hemamalini, S. Samshuddin, B. Narayana, H. S. Yathirajan

**Affiliations:** a X-ray Crystallography Unit, School of Physics, Universiti Sains Malaysia, 11800 USM, Penang, Malaysia; b Department of Studies in Chemistry, Mangalore University, Mangalagangotri 574 199, India; c Department of Studies in Chemistry, University of Mysore, Manasagangotri, Mysore 570 006, India

## Abstract

The 3-cyclo­hexene units adopt envelope conformations in each of the two independent mol­ecules that comprise the asymmetric unit of the title compound, C_20_H_16_F_2_O_3_. The dihedral angles between the two fluoro­phenyl rings are 79.7 (2) and 73.7 (2)° in the two mol­ecules. In one of the mol­ecules, two C—H groups of the cyclo­hexene ring are disordered over two sets of sites in a 0.818 (13):0.182 (13) ratio, the major and minor components corresponding to the two enanti­omeric forms of the mol­ecule. Weak inter­molecular C—H⋯O inter­actions help to stabilize the crystal structure.

## Related literature

For background to the applications of cyclo­hexenones, see: Padmavathi *et al.* (1999 ▶; 2000 ▶; 2001*a* ▶,*b* ▶); Hiromichi *et al.* (2002 ▶); Hoye & Tennakoon (2000 ▶); Kolesnick & Golde (1994 ▶); Tanaka *et al.* (1997 ▶). For related structures, see: Fischer *et al.* (2007*a* ▶,*b* ▶, 2008*a* ▶,*b* ▶); Li *et al.* (2009 ▶); Ashalatha *et al.* (2009 ▶); Sreevidya *et al.* (2010 ▶). For ring conformations, see: Cremer & Pople (1975 ▶).
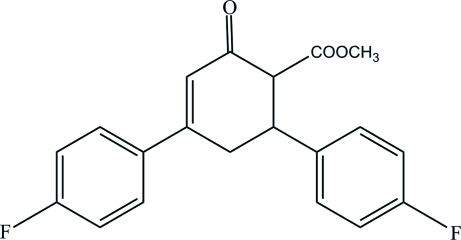


## Experimental

### 

#### Crystal data

C_20_H_16_F_2_O_3_
*M* *_r_* = 342.33
Orthorhombic, 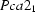

*a* = 17.3774 (5) Å
*b* = 9.0629 (3) Å
*c* = 22.2238 (7) Å
*V* = 3500.02 (19) Å^3^
*Z* = 8
Mo *K*α radiation
μ = 0.10 mm^−1^
*T* = 296 K
0.40 × 0.32 × 0.28 mm

#### Data collection

Bruker SMART APEXII CCD area-detector diffractometer
Absorption correction: multi-scan (*SADABS*; Bruker, 2009 ▶) *T*_min_ = 0.961, *T*_max_ = 0.973
38134 measured reflections
5278 independent reflections
2895 reflections with *I* > 2σ(*I*)
*R*_int_ = 0.065

#### Refinement

*R*[*F* ^2^ > 2σ(*F* ^2^)] = 0.055
*wR*(*F* ^2^) = 0.169
*S* = 1.02
5278 reflections
472 parameters
1 restraint
H-atom parameters constrained
Δρ_max_ = 0.17 e Å^−3^
Δρ_min_ = −0.14 e Å^−3^

### 

Data collection: *APEX2* (Bruker, 2009 ▶); cell refinement: *SAINT* (Bruker, 2009 ▶); data reduction: *SAINT*; program(s) used to solve structure: *SHELXTL* (Sheldrick, 2008 ▶); program(s) used to refine structure: *SHELXTL*; molecular graphics: *SHELXTL*; software used to prepare material for publication: *SHELXTL* and *PLATON* (Spek, 2009 ▶).

## Supplementary Material

Crystal structure: contains datablocks global, I. DOI: 10.1107/S1600536810009414/sj2744sup1.cif

Structure factors: contains datablocks I. DOI: 10.1107/S1600536810009414/sj2744Isup2.hkl

Additional supplementary materials:  crystallographic information; 3D view; checkCIF report

## Figures and Tables

**Table 1:** Hydrogen-bond geometry (Å, °)

*D*—H⋯*A*	*D*—H	H⋯*A*	*D*⋯*A*	*D*—H⋯*A*
C8*A*—H8*AB*⋯O2*B*^i^	0.97	2.38	3.263 (5)	151
C8*B*—H8*BB*⋯O2*A*^ii^	0.97	2.57	3.404 (5)	144
C15*B*—H15*B*⋯O1*B*^iii^	0.93	2.55	3.226 (6)	130

Symmetry codes: (i) 
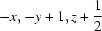
; (ii) 
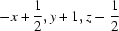
; (iii) 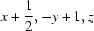.

**Fig. 1.**
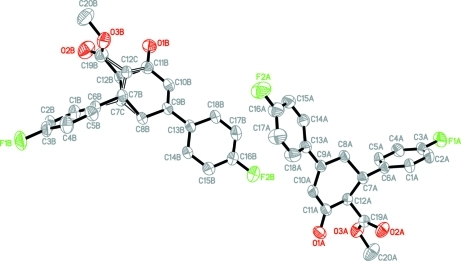
The asymmetric unit of the title compound, showing 30% probability displacement ellipsoids and the atom-numbering scheme. Bonds to atoms of the minor disorder component are drawn as open lines and H atoms are omitted for clarity.

**Fig. 2.**
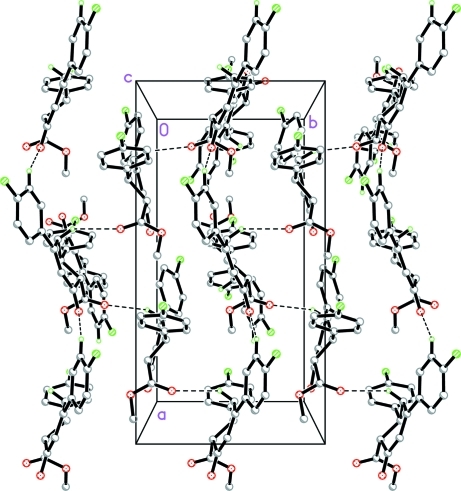
The crystal packing of the title compound, showing hydrogen-bonded network (dashed lines). H atoms not involved in hydrogen bond interactions and atoms of the minor disorder component are omitted for clarity.

## supplementary crystallographic information

### Comment

Cyclohexenones are efficient synthons for building spiro compounds (Padmavathi *et al.*, 2001*a*) or as intermediates in the synthesis of benzisoxazoles or carbazole derivatives (Padmavathi *et al.*, 1999; 2000; 2001*b*). Cyclohexenone derivatives are well known lead molecules for the treatment of inflammation and autoimmune diseases (Kolesnick & Golde, 1994; Tanaka *et al.*, 1997;  Hoye & Tennakoon, 2000; Hiromichi *et al.*, 2002).

The crystal structures of some cyclohexenone derivatives viz, (8RS,9SR)-ethyl 4-(3-bromothien-2-yl)-6-(2-furyl)-2-oxocyclohex-3- ene-1-carboxylate, (7RS,8SR)-ethyl 6-(1,3-benzodioxol-5-yl)-3-(3- bromo-2-thienyl)-2-oxocyclohex-3-ene-1-carboxylate, ethyl 4-(3-bromo -2-thienyl)-2-oxo-6-phenylcyclohex-3-ene-1-carboxylate,  rac-ethyl 3-(3-bromo-2-thienyl)-2-oxo-6-(4-propoxyphenyl)cyclohex-3-ene-1- carboxylate (Fischer *et al.*, 2007a,b; 2008a,b) and ethyl 6-(6-methoxy-2-naphthyl)-2-oxo-4-(2-thienyl)cyclohex-3-ene-1- carboxylate (Li *et al.*, 2009) have been reported. In a continuation of our work on cyclohexenone derivatives (Ashalatha *et al.*, 2009; Sreevidya *et al.*, 2010) and in view of the importance of these derivatives, the title compound (I) is synthesized and its crystal structure is reported here.

The asymmetric unit of the title compound consists of two crystallographically independent molecules, A and B (Fig. 1). The cyclohex-3-ene units in both molecules adopt envelope conformations with puckering parameters Q = 0.462 (4)Å, Θ = 128.3 (5)° and φ = 165.2 (6)° for molecule A and Q = 0.488 (6)Å, Θ = 128.5 (6)° and φ = 164.2 (7)° for molecule B (Cremer & Pople, 1975). The two fluorophenyl rings are inclined to each other forming dihedral angles of 79.7 (2)° (C1A–C6A:C13A–C18A) in molecule A and 73.7 (2)° (C1B–C6B:C13B–C18B) in molecule B.

The cyclohexene rings (C7–C12) are almost coplanar with C7A, C12A, C7B and C12B displaced by -0.322 (4)Å, 0.262 (4)Å, -0.339 (6)Å and 0.281 (5)Å. In molecule B, C7 and C12 are disordered over two sites with a 0.818 (13):0.182 (13) ratio and the minor disorder component shows displacements from the mean plane of C8B/C9B/C10B/C11B in the opposite sense to that of the major component: C7C = 0.33 (4)Å; C12C = -0.414 (18)Å. Atoms C7A, C12A, C7B and C12B are stereogenic centres. In molecule B, the major and minor disorder components correspond to the two enantiomeric forms of the molecule.

In the crystal packing (Fig. 2), the molecules are linked through weak intermolecular C8A—H8AB···O2B; C8B—H8BB···O2A and C15B—H15B···O1B hydrogen bonds (see Table 1).

### Experimental

A mixture of  (2E)-1,3-bis(4-fluorophenyl)prop-2-en-1-one (2.24g, 0.01mol) and methyl acetoacetate (0.01 mol) were refluxed for 2 hr in 10-15 ml of ethanol in presence of 0.8 ml 10% NaOH.  The reaction mixture was cooled to room temperature and the solid product obtained was filtered and recrystallized from toluene (M.P.: 417K).  Analytical data: found (calculated): C % : 70.08  (70.17); H% :  4.66 (4.71)

### Refinement

Atoms C7B and C12B and their attached H atoms are disordered over two sets of sites with a 0.818 (13):0.182 (13) occupancy ratio. All hydrogen atoms were positioned geometrically [C–H = 0.93 or 0.96Å] and were refined using a riding model, with *U*_iso_(H) = 1.2 or 1.5 *U*_eq_(C). A rotating group model was applied to the methyl groups. In the absence of large anomalous dispersion, 3128 Friedel pairs were merged for the final refinement.

### Crystal data

**Table:**

C_20_H_16_F_2_O_3_	*F*(000) = 1424
*M**_r_* = 342.33	*D*_x_ = 1.299 Mg m^−^^3^
Orthorhombic, *P**c**a*2_1_	Mo *K*α radiation, λ = 0.71073 Å
Hall symbol: P 2c -2ac	Cell parameters from 7809 reflections
*a* = 17.3774 (5) Å	θ = 2.5–21.9°
*b* = 9.0629 (3) Å	µ = 0.10 mm^−^^1^
*c* = 22.2238 (7) Å	*T* = 296 K
*V* = 3500.02 (19) Å^3^	Block, colourless
*Z* = 8	0.40 × 0.32 × 0.28 mm

### Data collection

**Table:**

Bruker SMART APEXII CCD area-detector diffractometer	5278 independent reflections
Radiation source: fine-focus sealed tube	2895 reflections with *I* > 2σ(*I*)
graphite	*R*_int_ = 0.065
φ and ω scans	θ_max_ = 30.2°, θ_min_ = 2.3°
Absorption correction: multi-scan (*SADABS*; Bruker, 2009)	*h* = −24→24
*T*_min_ = 0.961, *T*_max_ = 0.973	*k* = −12→12
38134 measured reflections	*l* = −20→31

### Refinement

**Table:**

Refinement on *F*^2^	Primary atom site location: structure-invariant direct methods
Least-squares matrix: full	Secondary atom site location: difference Fourier map
*R*[*F*^2^ > 2σ(*F*^2^)] = 0.055	Hydrogen site location: inferred from neighbouring sites
*wR*(*F*^2^) = 0.169	H-atom parameters constrained
*S* = 1.02	*w* = 1/[σ^2^(*F*_o_^2^) + (0.0821*P*)^2^ + 0.1221*P*] where *P* = (*F*_o_^2^ + 2*F*_c_^2^)/3
5278 reflections	(Δ/σ)_max_ = 0.001
472 parameters	Δρ_max_ = 0.17 e Å^−^^3^
1 restraint	Δρ_min_ = −0.14 e Å^−^^3^

### Special details

**Table:**

Geometry. All s.u.'s (except the s.u. in the dihedral angle between two l.s. planes) are estimated using the full covariance matrix. The cell s.u.'s are taken into account individually in the estimation of s.u.'s in distances, angles and torsion angles; correlations between s.u.'s in cell parameters are only used when they are defined by crystal symmetry. An approximate (isotropic) treatment of cell s.u.'s is used for estimating s.u.'s involving l.s. planes.
Refinement. Refinement of F^2^ against ALL reflections. The weighted R-factor wR and goodness of fit S are based on F^2^, conventional R-factors R are based on F, with F set to zero for negative F^2^. The threshold expression of F^2^ > 2σ(F^2^) is used only for calculating R-factors(gt) etc. and is not relevant to the choice of reflections for refinement. R-factors based on F^2^ are statistically about twice as large as those based on F, and R- factors based on ALL data will be even larger.

### Fractional atomic coordinates and isotropic or equivalent isotropic displacement parameters (Å^2^)

**Table:**

	*x*	*y*	*z*	*U*_iso_*/*U*_eq_	Occ. (<1)
F1A	0.13044 (18)	−0.1190 (4)	0.76691 (10)	0.1074 (9)	
F2A	−0.0040 (2)	−0.1767 (4)	0.23590 (14)	0.1323 (12)	
O1A	0.40617 (15)	0.0074 (3)	0.44238 (14)	0.0808 (8)	
O2A	0.39944 (18)	−0.1519 (3)	0.57537 (17)	0.0913 (9)	
O3A	0.41115 (18)	0.0919 (3)	0.57886 (15)	0.0886 (8)	
C1A	0.2085 (2)	−0.2420 (4)	0.62524 (16)	0.0726 (9)	
H1AA	0.2232	−0.3296	0.6067	0.087*	
C2A	0.1803 (3)	−0.2447 (5)	0.68369 (19)	0.0818 (11)	
H2AA	0.1762	−0.3329	0.7048	0.098*	
C3A	0.1588 (2)	−0.1153 (5)	0.70904 (16)	0.0751 (11)	
C4A	0.1641 (3)	0.0180 (5)	0.6812 (2)	0.0809 (11)	
H4AA	0.1496	0.1046	0.7006	0.097*	
C5A	0.1921 (3)	0.0189 (5)	0.62256 (19)	0.0763 (11)	
H5AA	0.1957	0.1080	0.6020	0.092*	
C6A	0.21478 (19)	−0.1097 (4)	0.59429 (14)	0.0601 (8)	
C7A	0.2441 (2)	−0.1129 (4)	0.52993 (16)	0.0615 (8)	
H7AA	0.2645	−0.2121	0.5226	0.074*	
C8A	0.1799 (2)	−0.0870 (4)	0.48469 (15)	0.0610 (8)	
H8AA	0.1432	−0.1673	0.4876	0.073*	
H8AB	0.1532	0.0035	0.4952	0.073*	
C9A	0.2075 (2)	−0.0759 (4)	0.42100 (15)	0.0599 (8)	
C10A	0.2816 (2)	−0.0426 (4)	0.40906 (16)	0.0650 (8)	
H10A	0.2969	−0.0383	0.3690	0.078*	
C11A	0.3381 (2)	−0.0134 (4)	0.45435 (17)	0.0640 (9)	
C12A	0.3102 (2)	−0.0035 (4)	0.51956 (16)	0.0611 (8)	
H12A	0.2909	0.0966	0.5267	0.073*	
C13A	0.1514 (2)	−0.1027 (4)	0.37197 (16)	0.0642 (8)	
C14A	0.0756 (2)	−0.0588 (4)	0.37815 (17)	0.0698 (9)	
H14A	0.0596	−0.0133	0.4135	0.084*	
C15A	0.0230 (2)	−0.0822 (5)	0.3320 (2)	0.0818 (11)	
H15A	−0.0279	−0.0517	0.3358	0.098*	
C16A	0.0481 (3)	−0.1514 (6)	0.2809 (2)	0.0956 (14)	
C17A	0.1221 (3)	−0.1967 (7)	0.2727 (2)	0.1112 (18)	
H17A	0.1371	−0.2429	0.2372	0.133*	
C18A	0.1741 (3)	−0.1723 (6)	0.3184 (2)	0.0933 (14)	
H18A	0.2250	−0.2022	0.3137	0.112*	
C19A	0.3777 (2)	−0.0302 (4)	0.56085 (17)	0.0634 (8)	
C20A	0.4811 (4)	0.0706 (6)	0.6153 (3)	0.124 (2)	
H20A	0.5034	0.1649	0.6246	0.187*	
H20B	0.5176	0.0126	0.5931	0.187*	
H20C	0.4681	0.0206	0.6520	0.187*	
F1B	0.1337 (2)	0.5160 (4)	−0.19775 (12)	0.1155 (10)	
F2B	0.2341 (2)	0.2462 (4)	0.32262 (14)	0.1301 (11)	
O1B	−0.15225 (18)	0.6114 (4)	0.12515 (15)	0.0933 (9)	
O2B	−0.1376 (3)	0.7363 (4)	−0.0131 (2)	0.1300 (15)	
O3B	−0.1543 (2)	0.4954 (3)	−0.00956 (17)	0.0966 (10)	
C1B	0.0509 (2)	0.6346 (5)	−0.05757 (19)	0.0760 (10)	
H1BA	0.0377	0.7220	−0.0382	0.091*	
C2B	0.0835 (2)	0.6400 (5)	−0.11435 (18)	0.0781 (11)	
H2BA	0.0924	0.7299	−0.1333	0.094*	
C3B	0.1021 (3)	0.5116 (5)	−0.14166 (17)	0.0788 (11)	
C4B	0.0910 (3)	0.3776 (5)	−0.1145 (2)	0.0861 (12)	
H4BA	0.1053	0.2906	−0.1337	0.103*	
C5B	0.0582 (3)	0.3747 (5)	−0.0583 (2)	0.0793 (11)	
H5BA	0.0498	0.2842	−0.0397	0.095*	
C6B	0.0378 (2)	0.5006 (4)	−0.02920 (16)	0.0640 (9)	
C7B	0.0036 (3)	0.4823 (7)	0.0331 (3)	0.0566 (14)	0.818 (13)
H7BA	−0.0238	0.3878	0.0343	0.068*	0.818 (13)
C7C	0.0136 (17)	0.552 (4)	0.0413 (11)	0.063 (7)	0.182 (13)
H7CA	0.0175	0.6596	0.0457	0.076*	0.182 (13)
C8B	0.06828 (19)	0.4779 (4)	0.08113 (15)	0.0580 (8)	
H8BA	0.1001	0.3917	0.0741	0.070*	
H8BB	0.1006	0.5645	0.0763	0.070*	
C9B	0.0387 (2)	0.4732 (4)	0.14515 (16)	0.0600 (8)	
C10B	−0.0333 (2)	0.5192 (4)	0.15751 (17)	0.0681 (9)	
H10B	−0.0503	0.5133	0.1971	0.082*	
C11B	−0.0858 (2)	0.5770 (5)	0.11294 (19)	0.0733 (10)	
C12B	−0.0537 (3)	0.6048 (6)	0.0490 (2)	0.0618 (16)	0.818 (13)
H12B	−0.0253	0.6982	0.0499	0.074*	0.818 (13)
C12C	−0.0661 (11)	0.504 (2)	0.0472 (9)	0.056 (6)	0.182 (13)
H12C	−0.0764	0.3988	0.0421	0.067*	0.182 (13)
C13B	0.0905 (2)	0.4144 (4)	0.19204 (16)	0.0652 (8)	
C14B	0.1692 (2)	0.4082 (4)	0.18360 (18)	0.0715 (9)	
H14B	0.1900	0.4412	0.1475	0.086*	
C15B	0.2173 (3)	0.3544 (5)	0.2275 (2)	0.0835 (11)	
H15B	0.2702	0.3523	0.2213	0.100*	
C16B	0.1867 (3)	0.3042 (6)	0.2801 (2)	0.0904 (13)	
C17B	0.1109 (3)	0.3063 (8)	0.2898 (2)	0.118 (2)	
H17B	0.0911	0.2706	0.3258	0.142*	
C18B	0.0621 (3)	0.3607 (7)	0.2468 (2)	0.1047 (17)	
H18B	0.0094	0.3620	0.2541	0.126*	
C19B	−0.1188 (3)	0.6222 (6)	0.0055 (2)	0.0869 (13)	
C20B	−0.2189 (4)	0.5075 (7)	−0.0513 (4)	0.135 (2)	
H20D	−0.2467	0.4160	−0.0520	0.202*	
H20E	−0.1998	0.5289	−0.0909	0.202*	
H20F	−0.2525	0.5854	−0.0385	0.202*	

### Atomic displacement parameters (Å^2^)

**Table:**

	*U*^11^	*U*^22^	*U*^33^	*U*^12^	*U*^13^	*U*^23^
F1A	0.116 (2)	0.162 (3)	0.0439 (12)	−0.0081 (18)	0.0163 (13)	0.0076 (14)
F2A	0.108 (2)	0.193 (3)	0.0960 (19)	−0.007 (2)	−0.0281 (18)	−0.033 (2)
O1A	0.0687 (16)	0.0973 (19)	0.0764 (18)	−0.0123 (13)	0.0137 (14)	0.0075 (14)
O2A	0.104 (2)	0.0625 (15)	0.108 (2)	0.0139 (14)	−0.0235 (19)	0.0038 (16)
O3A	0.105 (2)	0.0661 (15)	0.095 (2)	0.0003 (14)	−0.0244 (18)	−0.0034 (15)
C1A	0.090 (2)	0.070 (2)	0.058 (2)	−0.0041 (19)	0.0068 (18)	0.0089 (17)
C2A	0.100 (3)	0.087 (3)	0.059 (2)	−0.009 (2)	0.004 (2)	0.017 (2)
C3A	0.075 (2)	0.109 (3)	0.0412 (17)	−0.015 (2)	0.0014 (16)	0.0091 (19)
C4A	0.092 (3)	0.086 (3)	0.065 (2)	−0.002 (2)	0.017 (2)	−0.010 (2)
C5A	0.098 (3)	0.070 (2)	0.061 (2)	−0.0042 (19)	0.011 (2)	0.0109 (18)
C6A	0.065 (2)	0.068 (2)	0.0467 (17)	−0.0039 (15)	0.0049 (15)	0.0042 (15)
C7A	0.069 (2)	0.0625 (17)	0.0533 (18)	0.0012 (15)	0.0074 (16)	0.0019 (15)
C8A	0.064 (2)	0.0695 (19)	0.0497 (17)	−0.0039 (16)	0.0066 (15)	0.0044 (16)
C9A	0.064 (2)	0.0624 (18)	0.0537 (19)	0.0051 (15)	0.0086 (15)	0.0026 (15)
C10A	0.072 (2)	0.076 (2)	0.0466 (17)	0.0029 (16)	0.0103 (16)	0.0098 (16)
C11A	0.069 (2)	0.0607 (18)	0.062 (2)	0.0013 (15)	0.0136 (18)	0.0079 (16)
C12A	0.069 (2)	0.0587 (18)	0.055 (2)	0.0046 (14)	0.0013 (16)	0.0045 (14)
C13A	0.065 (2)	0.078 (2)	0.0495 (17)	−0.0001 (16)	0.0061 (15)	−0.0003 (16)
C14A	0.072 (2)	0.084 (2)	0.053 (2)	0.0041 (18)	0.0064 (17)	−0.0032 (18)
C15A	0.070 (2)	0.104 (3)	0.071 (2)	0.003 (2)	0.002 (2)	0.002 (2)
C16A	0.084 (3)	0.129 (4)	0.073 (3)	−0.009 (3)	−0.011 (2)	−0.019 (3)
C17A	0.100 (4)	0.166 (5)	0.068 (3)	0.010 (3)	−0.004 (3)	−0.040 (3)
C18A	0.076 (3)	0.132 (4)	0.071 (3)	0.009 (2)	0.009 (2)	−0.026 (3)
C19A	0.072 (2)	0.0587 (19)	0.059 (2)	0.0005 (15)	0.0010 (17)	−0.0020 (16)
C20A	0.136 (5)	0.101 (3)	0.136 (5)	−0.009 (3)	−0.069 (4)	−0.003 (3)
F1B	0.147 (3)	0.147 (2)	0.0525 (13)	0.013 (2)	0.0230 (15)	0.0068 (14)
F2B	0.118 (2)	0.176 (3)	0.0961 (19)	0.005 (2)	−0.0205 (18)	0.050 (2)
O1B	0.0784 (19)	0.112 (2)	0.090 (2)	0.0279 (16)	0.0214 (16)	0.0041 (17)
O2B	0.147 (3)	0.084 (2)	0.158 (4)	0.013 (2)	−0.034 (3)	0.021 (2)
O3B	0.115 (2)	0.084 (2)	0.092 (2)	0.0142 (17)	−0.0098 (19)	0.0189 (16)
C1B	0.094 (3)	0.074 (2)	0.060 (2)	−0.0007 (19)	0.006 (2)	0.0019 (18)
C2B	0.100 (3)	0.077 (2)	0.057 (2)	−0.015 (2)	0.004 (2)	0.0165 (19)
C3B	0.095 (3)	0.098 (3)	0.0426 (18)	−0.009 (2)	0.0042 (19)	0.0066 (18)
C4B	0.111 (3)	0.082 (3)	0.065 (2)	0.002 (2)	0.004 (2)	−0.014 (2)
C5B	0.092 (3)	0.070 (2)	0.076 (3)	−0.0006 (19)	0.014 (2)	0.0053 (19)
C6B	0.070 (2)	0.071 (2)	0.0512 (19)	−0.0014 (16)	0.0025 (16)	0.0074 (16)
C7B	0.073 (3)	0.044 (3)	0.053 (3)	−0.003 (2)	0.007 (2)	0.001 (2)
C7C	0.081 (16)	0.066 (15)	0.042 (10)	−0.010 (14)	−0.001 (9)	−0.008 (12)
C8B	0.0652 (19)	0.0545 (15)	0.0542 (19)	−0.0027 (13)	0.0111 (16)	0.0033 (14)
C9B	0.069 (2)	0.0576 (17)	0.0533 (18)	−0.0107 (15)	0.0103 (16)	0.0011 (15)
C10B	0.075 (2)	0.073 (2)	0.055 (2)	−0.0031 (17)	0.0146 (17)	0.0001 (17)
C11B	0.076 (3)	0.077 (2)	0.067 (2)	0.0070 (19)	0.010 (2)	−0.0035 (19)
C12B	0.069 (3)	0.053 (3)	0.063 (3)	0.005 (2)	0.015 (2)	0.0016 (19)
C12C	0.072 (13)	0.036 (12)	0.061 (11)	−0.001 (8)	0.002 (9)	0.008 (7)
C13B	0.073 (2)	0.070 (2)	0.0530 (18)	−0.0125 (16)	0.0081 (17)	0.0045 (16)
C14B	0.073 (2)	0.089 (2)	0.0526 (18)	−0.0039 (19)	0.0089 (17)	0.0108 (18)
C15B	0.078 (3)	0.102 (3)	0.070 (3)	0.001 (2)	0.004 (2)	0.009 (2)
C16B	0.089 (3)	0.110 (3)	0.072 (3)	−0.001 (2)	−0.010 (2)	0.025 (2)
C17B	0.107 (4)	0.171 (6)	0.076 (3)	−0.018 (4)	0.000 (3)	0.052 (3)
C18B	0.084 (3)	0.158 (5)	0.073 (3)	−0.014 (3)	0.009 (2)	0.046 (3)
C19B	0.089 (3)	0.085 (3)	0.087 (3)	0.014 (2)	0.004 (2)	0.006 (2)
C20B	0.134 (5)	0.130 (4)	0.142 (6)	−0.020 (4)	−0.060 (5)	0.029 (4)

### Geometric parameters (Å, °)

**Table:**

F1A—C3A	1.378 (4)	O3B—C19B	1.346 (6)
F2A—C16A	1.368 (5)	O3B—C20B	1.461 (7)
O1A—C11A	1.227 (4)	C1B—C2B	1.384 (6)
O2A—C19A	1.210 (4)	C1B—C6B	1.388 (6)
O3A—C19A	1.312 (4)	C1B—H1BA	0.9300
O3A—C20A	1.474 (6)	C2B—C3B	1.352 (6)
C1A—C6A	1.387 (5)	C2B—H2BA	0.9300
C1A—C2A	1.388 (6)	C3B—C4B	1.370 (7)
C1A—H1AA	0.9300	C4B—C5B	1.373 (6)
C2A—C3A	1.353 (7)	C4B—H4BA	0.9300
C2A—H2AA	0.9300	C5B—C6B	1.359 (6)
C3A—C4A	1.360 (6)	C5B—H5BA	0.9300
C4A—C5A	1.392 (6)	C6B—C7B	1.516 (6)
C4A—H4AA	0.9300	C6B—C7C	1.69 (3)
C5A—C6A	1.381 (6)	C7B—C12B	1.532 (8)
C5A—H5AA	0.9300	C7B—C8B	1.551 (7)
C6A—C7A	1.519 (5)	C7B—H7BA	0.9800
C7A—C8A	1.521 (5)	C7C—C12C	1.46 (4)
C7A—C12A	1.535 (5)	C7C—C8B	1.46 (3)
C7A—H7AA	0.9800	C7C—H7CA	0.9800
C8A—C9A	1.498 (5)	C8B—C9B	1.514 (5)
C8A—H8AA	0.9700	C8B—H8BA	0.9700
C8A—H8AB	0.9700	C8B—H8BB	0.9700
C9A—C10A	1.350 (5)	C9B—C10B	1.346 (5)
C9A—C13A	1.482 (5)	C9B—C13B	1.477 (5)
C10A—C11A	1.430 (5)	C10B—C11B	1.445 (6)
C10A—H10A	0.9300	C10B—H10B	0.9300
C11A—C12A	1.531 (5)	C11B—C12B	1.548 (6)
C12A—C19A	1.509 (5)	C11B—C12C	1.64 (2)
C12A—H12A	0.9800	C12B—C19B	1.496 (7)
C13A—C14A	1.383 (5)	C12B—H12B	0.9800
C13A—C18A	1.404 (6)	C12C—C19B	1.685 (19)
C14A—C15A	1.389 (6)	C12C—H12C	0.9800
C14A—H14A	0.9300	C13B—C14B	1.380 (5)
C15A—C16A	1.370 (7)	C13B—C18B	1.401 (6)
C15A—H15A	0.9300	C14B—C15B	1.374 (6)
C16A—C17A	1.362 (7)	C14B—H14B	0.9300
C17A—C18A	1.378 (7)	C15B—C16B	1.363 (6)
C17A—H17A	0.9300	C15B—H15B	0.9300
C18A—H18A	0.9300	C16B—C17B	1.335 (7)
C20A—H20A	0.9600	C17B—C18B	1.368 (7)
C20A—H20B	0.9600	C17B—H17B	0.9300
C20A—H20C	0.9600	C18B—H18B	0.9300
F1B—C3B	1.363 (5)	C20B—H20D	0.9600
F2B—C16B	1.359 (5)	C20B—H20E	0.9600
O1B—C11B	1.227 (5)	C20B—H20F	0.9600
O2B—C19B	1.160 (5)		
			
C19A—O3A—C20A	115.0 (3)	C6B—C5B—C4B	121.7 (4)
C6A—C1A—C2A	120.5 (4)	C6B—C5B—H5BA	119.1
C6A—C1A—H1AA	119.8	C4B—C5B—H5BA	119.1
C2A—C1A—H1AA	119.8	C5B—C6B—C1B	118.4 (4)
C3A—C2A—C1A	118.2 (4)	C5B—C6B—C7B	116.5 (4)
C3A—C2A—H2AA	120.9	C1B—C6B—C7B	125.1 (4)
C1A—C2A—H2AA	120.9	C5B—C6B—C7C	137.7 (12)
C2A—C3A—C4A	124.2 (4)	C1B—C6B—C7C	102.7 (12)
C2A—C3A—F1A	117.8 (4)	C7B—C6B—C7C	23.8 (10)
C4A—C3A—F1A	118.0 (4)	C6B—C7B—C12B	112.7 (4)
C3A—C4A—C5A	117.0 (4)	C6B—C7B—C8B	110.3 (4)
C3A—C4A—H4AA	121.5	C12B—C7B—C8B	109.4 (5)
C5A—C4A—H4AA	121.5	C6B—C7B—H7BA	108.1
C6A—C5A—C4A	121.4 (4)	C12B—C7B—H7BA	108.1
C6A—C5A—H5AA	119.3	C8B—C7B—H7BA	108.1
C4A—C5A—H5AA	119.3	C12C—C7C—C8B	115 (2)
C5A—C6A—C1A	118.8 (3)	C12C—C7C—C6B	103.7 (19)
C5A—C6A—C7A	122.7 (3)	C8B—C7C—C6B	105.8 (18)
C1A—C6A—C7A	118.5 (3)	C12C—C7C—H7CA	110.6
C6A—C7A—C8A	111.9 (3)	C8B—C7C—H7CA	110.6
C6A—C7A—C12A	112.4 (3)	C6B—C7C—H7CA	110.6
C8A—C7A—C12A	110.5 (3)	C7C—C8B—C9B	111.2 (10)
C6A—C7A—H7AA	107.3	C7C—C8B—C7B	26.0 (12)
C8A—C7A—H7AA	107.3	C9B—C8B—C7B	113.6 (3)
C12A—C7A—H7AA	107.3	C7C—C8B—H8BA	130.1
C9A—C8A—C7A	113.6 (3)	C9B—C8B—H8BA	108.8
C9A—C8A—H8AA	108.8	C7B—C8B—H8BA	108.8
C7A—C8A—H8AA	108.8	C7C—C8B—H8BB	86.3
C9A—C8A—H8AB	108.8	C9B—C8B—H8BB	108.8
C7A—C8A—H8AB	108.8	C7B—C8B—H8BB	108.8
H8AA—C8A—H8AB	107.7	H8BA—C8B—H8BB	107.7
C10A—C9A—C13A	121.3 (3)	C10B—C9B—C13B	122.3 (3)
C10A—C9A—C8A	120.4 (3)	C10B—C9B—C8B	119.9 (3)
C13A—C9A—C8A	118.3 (3)	C13B—C9B—C8B	117.8 (3)
C9A—C10A—C11A	123.9 (3)	C9B—C10B—C11B	124.0 (3)
C9A—C10A—H10A	118.0	C9B—C10B—H10B	118.0
C11A—C10A—H10A	118.0	C11B—C10B—H10B	118.0
O1A—C11A—C10A	122.5 (3)	O1B—C11B—C10B	122.3 (4)
O1A—C11A—C12A	120.1 (4)	O1B—C11B—C12B	120.0 (4)
C10A—C11A—C12A	117.4 (3)	C10B—C11B—C12B	117.5 (3)
C19A—C12A—C11A	108.7 (3)	O1B—C11B—C12C	119.8 (8)
C19A—C12A—C7A	112.7 (3)	C10B—C11B—C12C	109.5 (7)
C11A—C12A—C7A	109.9 (3)	C12B—C11B—C12C	34.0 (7)
C19A—C12A—H12A	108.5	C19B—C12B—C7B	114.8 (5)
C11A—C12A—H12A	108.5	C19B—C12B—C11B	109.7 (4)
C7A—C12A—H12A	108.5	C7B—C12B—C11B	109.1 (4)
C14A—C13A—C18A	118.8 (4)	C19B—C12B—H12B	107.7
C14A—C13A—C9A	120.4 (3)	C7B—C12B—H12B	107.7
C18A—C13A—C9A	120.8 (3)	C11B—C12B—H12B	107.7
C13A—C14A—C15A	120.6 (4)	C7C—C12C—C11B	99.1 (17)
C13A—C14A—H14A	119.7	C7C—C12C—C19B	106.2 (19)
C15A—C14A—H14A	119.7	C11B—C12C—C19B	97.0 (11)
C16A—C15A—C14A	118.2 (4)	C7C—C12C—H12C	117.1
C16A—C15A—H15A	120.9	C11B—C12C—H12C	117.1
C14A—C15A—H15A	120.9	C19B—C12C—H12C	117.1
C17A—C16A—F2A	118.5 (4)	C14B—C13B—C18B	116.9 (4)
C17A—C16A—C15A	123.3 (4)	C14B—C13B—C9B	121.5 (3)
F2A—C16A—C15A	118.2 (4)	C18B—C13B—C9B	121.6 (4)
C16A—C17A—C18A	118.2 (4)	C15B—C14B—C13B	121.4 (4)
C16A—C17A—H17A	120.9	C15B—C14B—H14B	119.3
C18A—C17A—H17A	120.9	C13B—C14B—H14B	119.3
C17A—C18A—C13A	120.8 (4)	C16B—C15B—C14B	119.4 (4)
C17A—C18A—H18A	119.6	C16B—C15B—H15B	120.3
C13A—C18A—H18A	119.6	C14B—C15B—H15B	120.3
O2A—C19A—O3A	123.3 (4)	C17B—C16B—F2B	119.5 (4)
O2A—C19A—C12A	123.4 (3)	C17B—C16B—C15B	121.2 (4)
O3A—C19A—C12A	113.2 (3)	F2B—C16B—C15B	119.3 (4)
O3A—C20A—H20A	109.5	C16B—C17B—C18B	120.3 (4)
O3A—C20A—H20B	109.5	C16B—C17B—H17B	119.9
H20A—C20A—H20B	109.5	C18B—C17B—H17B	119.9
O3A—C20A—H20C	109.5	C17B—C18B—C13B	120.9 (4)
H20A—C20A—H20C	109.5	C17B—C18B—H18B	119.5
H20B—C20A—H20C	109.5	C13B—C18B—H18B	119.5
C19B—O3B—C20B	116.5 (4)	O2B—C19B—O3B	122.9 (5)
C2B—C1B—C6B	120.8 (4)	O2B—C19B—C12B	122.4 (5)
C2B—C1B—H1BA	119.6	O3B—C19B—C12B	114.6 (4)
C6B—C1B—H1BA	119.6	O2B—C19B—C12C	156.0 (9)
C3B—C2B—C1B	118.5 (4)	O3B—C19B—C12C	81.1 (8)
C3B—C2B—H2BA	120.8	C12B—C19B—C12C	33.6 (7)
C1B—C2B—H2BA	120.8	O3B—C20B—H20D	109.5
C2B—C3B—F1B	118.8 (4)	O3B—C20B—H20E	109.5
C2B—C3B—C4B	122.1 (4)	H20D—C20B—H20E	109.5
F1B—C3B—C4B	119.1 (4)	O3B—C20B—H20F	109.5
C3B—C4B—C5B	118.4 (4)	H20D—C20B—H20F	109.5
C3B—C4B—H4BA	120.8	H20E—C20B—H20F	109.5
C5B—C4B—H4BA	120.8		
			
C6A—C1A—C2A—C3A	0.5 (6)	C7B—C6B—C7C—C12C	−46 (2)
C1A—C2A—C3A—C4A	−0.9 (7)	C5B—C6B—C7C—C8B	43 (3)
C1A—C2A—C3A—F1A	179.6 (4)	C1B—C6B—C7C—C8B	−123.0 (17)
C2A—C3A—C4A—C5A	1.2 (7)	C7B—C6B—C7C—C8B	75 (3)
F1A—C3A—C4A—C5A	−179.3 (4)	C12C—C7C—C8B—C9B	−48 (3)
C3A—C4A—C5A—C6A	−1.0 (7)	C6B—C7C—C8B—C9B	−161.5 (11)
C4A—C5A—C6A—C1A	0.6 (6)	C12C—C7C—C8B—C7B	53 (3)
C4A—C5A—C6A—C7A	178.8 (4)	C6B—C7C—C8B—C7B	−61 (2)
C2A—C1A—C6A—C5A	−0.3 (6)	C6B—C7B—C8B—C7C	85 (3)
C2A—C1A—C6A—C7A	−178.5 (4)	C12B—C7B—C8B—C7C	−40 (3)
C5A—C6A—C7A—C8A	−72.1 (5)	C6B—C7B—C8B—C9B	175.0 (3)
C1A—C6A—C7A—C8A	106.0 (4)	C12B—C7B—C8B—C9B	50.5 (5)
C5A—C6A—C7A—C12A	52.9 (5)	C7C—C8B—C9B—C10B	7.0 (15)
C1A—C6A—C7A—C12A	−128.9 (4)	C7B—C8B—C9B—C10B	−21.0 (5)
C6A—C7A—C8A—C9A	174.9 (3)	C7C—C8B—C9B—C13B	−174.2 (15)
C12A—C7A—C8A—C9A	48.8 (4)	C7B—C8B—C9B—C13B	157.7 (4)
C7A—C8A—C9A—C10A	−20.7 (4)	C13B—C9B—C10B—C11B	−179.9 (4)
C7A—C8A—C9A—C13A	159.1 (3)	C8B—C9B—C10B—C11B	−1.2 (5)
C13A—C9A—C10A—C11A	179.0 (3)	C9B—C10B—C11B—O1B	176.5 (4)
C8A—C9A—C10A—C11A	−1.1 (5)	C9B—C10B—C11B—C12B	−7.6 (6)
C9A—C10A—C11A—O1A	175.3 (4)	C9B—C10B—C11B—C12C	28.6 (9)
C9A—C10A—C11A—C12A	−6.8 (5)	C6B—C7B—C12B—C19B	56.2 (7)
O1A—C11A—C12A—C19A	−23.2 (4)	C8B—C7B—C12B—C19B	179.2 (4)
C10A—C11A—C12A—C19A	158.8 (3)	C6B—C7B—C12B—C11B	179.8 (4)
O1A—C11A—C12A—C7A	−147.0 (3)	C8B—C7B—C12B—C11B	−57.2 (6)
C10A—C11A—C12A—C7A	35.0 (4)	O1B—C11B—C12B—C19B	−20.0 (6)
C6A—C7A—C12A—C19A	57.7 (4)	C10B—C11B—C12B—C19B	163.9 (4)
C8A—C7A—C12A—C19A	−176.5 (3)	C12C—C11B—C12B—C19B	79.6 (13)
C6A—C7A—C12A—C11A	179.2 (3)	O1B—C11B—C12B—C7B	−146.6 (5)
C8A—C7A—C12A—C11A	−55.1 (4)	C10B—C11B—C12B—C7B	37.4 (6)
C10A—C9A—C13A—C14A	−144.8 (4)	C12C—C11B—C12B—C7B	−47.0 (12)
C8A—C9A—C13A—C14A	35.4 (5)	C8B—C7C—C12C—C11B	71 (2)
C10A—C9A—C13A—C18A	35.0 (6)	C6B—C7C—C12C—C11B	−173.6 (13)
C8A—C9A—C13A—C18A	−144.9 (4)	C8B—C7C—C12C—C19B	171.4 (15)
C18A—C13A—C14A—C15A	−0.5 (6)	C6B—C7C—C12C—C19B	−74 (2)
C9A—C13A—C14A—C15A	179.3 (4)	O1B—C11B—C12C—C7C	152.4 (16)
C13A—C14A—C15A—C16A	0.9 (7)	C10B—C11B—C12C—C7C	−58.7 (19)
C14A—C15A—C16A—C17A	−0.8 (8)	C12B—C11B—C12C—C7C	51.8 (17)
C14A—C15A—C16A—F2A	178.9 (4)	O1B—C11B—C12C—C19B	44.7 (12)
F2A—C16A—C17A—C18A	−179.4 (6)	C10B—C11B—C12C—C19B	−166.5 (6)
C15A—C16A—C17A—C18A	0.4 (10)	C12B—C11B—C12C—C19B	−55.9 (9)
C16A—C17A—C18A—C13A	0.1 (9)	C10B—C9B—C13B—C14B	−160.8 (4)
C14A—C13A—C18A—C17A	0.0 (8)	C8B—C9B—C13B—C14B	20.5 (5)
C9A—C13A—C18A—C17A	−179.7 (5)	C10B—C9B—C13B—C18B	20.0 (6)
C20A—O3A—C19A—O2A	3.9 (7)	C8B—C9B—C13B—C18B	−158.7 (4)
C20A—O3A—C19A—C12A	−175.3 (5)	C18B—C13B—C14B—C15B	−1.2 (7)
C11A—C12A—C19A—O2A	−84.5 (4)	C9B—C13B—C14B—C15B	179.5 (4)
C7A—C12A—C19A—O2A	37.7 (5)	C13B—C14B—C15B—C16B	0.9 (7)
C11A—C12A—C19A—O3A	94.8 (4)	C14B—C15B—C16B—C17B	0.0 (8)
C7A—C12A—C19A—O3A	−143.1 (3)	C14B—C15B—C16B—F2B	177.7 (4)
C6B—C1B—C2B—C3B	0.1 (7)	F2B—C16B—C17B—C18B	−178.3 (6)
C1B—C2B—C3B—F1B	179.5 (4)	C15B—C16B—C17B—C18B	−0.6 (10)
C1B—C2B—C3B—C4B	−1.2 (7)	C16B—C17B—C18B—C13B	0.3 (10)
C2B—C3B—C4B—C5B	1.5 (7)	C14B—C13B—C18B—C17B	0.6 (8)
F1B—C3B—C4B—C5B	−179.2 (4)	C9B—C13B—C18B—C17B	179.9 (5)
C3B—C4B—C5B—C6B	−0.8 (7)	C20B—O3B—C19B—O2B	0.0 (8)
C4B—C5B—C6B—C1B	−0.1 (7)	C20B—O3B—C19B—C12B	179.3 (5)
C4B—C5B—C6B—C7B	−178.9 (5)	C20B—O3B—C19B—C12C	179.3 (8)
C4B—C5B—C6B—C7C	−165.1 (15)	C7B—C12B—C19B—O2B	−132.9 (6)
C2B—C1B—C6B—C5B	0.5 (6)	C11B—C12B—C19B—O2B	103.9 (6)
C2B—C1B—C6B—C7B	179.1 (4)	C7B—C12B—C19B—O3B	47.9 (6)
C2B—C1B—C6B—C7C	170.2 (10)	C11B—C12B—C19B—O3B	−75.4 (5)
C5B—C6B—C7B—C12B	−150.1 (5)	C7B—C12B—C19B—C12C	47.8 (13)
C1B—C6B—C7B—C12B	31.3 (7)	C11B—C12B—C19B—C12C	−75.5 (13)
C7C—C6B—C7B—C12B	53 (2)	C7C—C12C—C19B—O2B	−43 (3)
C5B—C6B—C7B—C8B	87.4 (5)	C11B—C12C—C19B—O2B	59 (2)
C1B—C6B—C7B—C8B	−91.2 (5)	C7C—C12C—C19B—O3B	138.5 (18)
C7C—C6B—C7B—C8B	−69 (3)	C11B—C12C—C19B—O3B	−119.8 (9)
C5B—C6B—C7C—C12C	−78 (2)	C7C—C12C—C19B—C12B	−41.6 (15)
C1B—C6B—C7C—C12C	115 (2)	C11B—C12C—C19B—C12B	60.1 (10)

### Hydrogen-bond geometry (Å, °)

**Table:**

*D*—H···*A*	*D*—H	H···*A*	*D*···*A*	*D*—H···*A*
C8A—H8AB···O2B^i^	0.97	2.38	3.263 (5)	151
C8B—H8BB···O2A^ii^	0.97	2.57	3.404 (5)	144
C15B—H15B···O1B^iii^	0.93	2.55	3.226 (6)	130

Symmetry codes: (i) −*x*, −*y*+1, *z*+1/2; (ii) −*x*+1/2, *y*+1, *z*−1/2; (iii) *x*+1/2, −*y*+1, *z*.
